# Compression Osteosynthesis Without Iliac Crest Osteotomy Through the Anterior Iliac Approach for Incomplete High Anterior Column Fractures of the Acetabulum: A Case Series and Surgical Technique

**DOI:** 10.3390/jcm15072739

**Published:** 2026-04-04

**Authors:** Young-Ho Cho, Young-Soo Byun, Seong-Eun Byun

**Affiliations:** 1 Department of Orthopaedic Surgery, Daegu Fatima Hospital, Daegu 41199, Republic of Korea; femur1973@hanmail.net (Y.-H.C.); byun0441@gmail.com (Y.-S.B.); 2 Department of Orthopedic Surgery, Burjeel Hospital Abu Dhabi, Abu Dhabi P.O. Box 7400, United Arab Emirates

**Keywords:** acetabulum, high anterior column fracture, anterior iliac approach, compression osteosynthesis

## Abstract

**Introduction:** An incomplete high anterior column fracture of the acetabulum is commonly considered to require completion of the fracture. However, reduction may become more difficult after completing the incomplete fracture due to plastic deformation. This study describes a surgical technique of compression osteosynthesis without completing the incomplete fracture and evaluates the clinical and radiographic outcomes. **Materials and Methods:** In this retrospective study, 25 patients with incomplete high anterior column fractures met the inclusion criteria. The fracture was reduced and stabilized by compression osteosynthesis through the anterior iliac approach without completing the incomplete fracture in the iliac wing. Patient demographics, the mechanism of injury, associated injuries, time to surgical reconstruction, operation time, and postoperative complications were analyzed. The quality of reduction and outcome were evaluated according to Matta’s criteria. **Results:** The mean operation time was 110 ± 23 min (range, 75–160). All fractures achieved bone union at a mean of 10.2 ± 1.4 weeks (range, 8–14). The quality of fracture reduction was graded as anatomical in 22 hips, imperfect in one and poor in two. Clinical results were excellent in 19 patients and good in six, and radiographic results were excellent in 22 patients and good in three. No statistically significant differences were observed between patients with and without quadrilateral plate fractures. Lateral femoral cutaneous nerve injury occurred in 13 patients (52%), mostly without significant symptoms. One patient experienced vascular injury. **Conclusions:** Incomplete high anterior column fractures can be effectively reduced and stabilized by compression osteosynthesis through the anterior iliac approach without completing the incomplete fracture in the iliac wing. This case series demonstrated favorable clinical and radiographic outcomes using this surgical technique. However, because this study was a retrospective case series with a small sample size and no comparative control group, further studies are required to confirm these findings.

## 1. Introduction

Isolated anterior column fractures of the acetabulum are rare injuries that account for 3–6.3% of total acetabular fractures [1,2,3]. However, the incidence of anterior column fractures has increased, particularly in older patients secondary to low-energy injuries in recent years [4,5]. Displaced anterior column fractures have increasingly been treated surgically by open reduction and internal fixation.

In high anterior column fractures of the acetabulum, occasionally, the fracture is incomplete, with the fracture line not exiting the iliac crest. In this fracture pattern, surgeons commonly try to achieve anatomic reduction in the anterior column by converting the incomplete iliac crest fracture into a complete fracture through osteotomy [6,7]. However, in such cases, plastic deformation of the fragment may occur, and even after osteotomy, achieving anatomic reduction can be difficult. This represents a key limitation of the conventional approach and poses a challenge in surgical decision-making. Therefore, avoiding osteotomy may be beneficial in this fracture subtype. However, there is currently a lack of clinical evidence regarding surgical techniques that preserve the incomplete iliac crest. To address this issue, we have applied a surgical technique of compression osteosynthesis without osteotomizing the incomplete iliac crest fracture using a minimally invasive approach.

The current study aimed to identify the usefulness of this novel method by evaluating the clinical and radiographic outcomes of this novel surgical technique in displaced incomplete high anterior column fractures.

### 1.1. Patients and Methods

**Figure 1:**
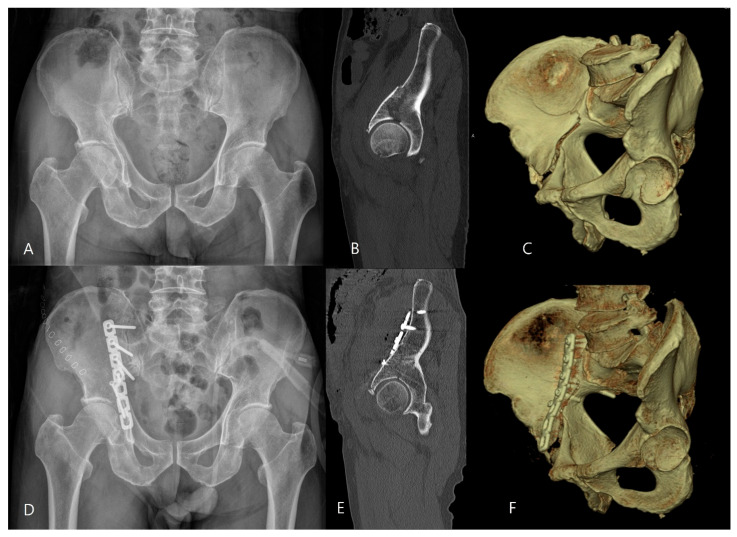
Preoperative images. (**A**) Anteroposterior radiograph of a right acetabular fracture, (**B**) a sagittal reconstruction of computed tomography (CT) scan (**B**), and three-dimensional CT scan (**C**) showing an incomplete anterior column fracture not involving the quadrilateral plate. Postoperative anteroposterior radiograph (**D**) of the anterior column fracture stabilized with a 4.5 mm reconstruction plate. Postoperative sagittal reconstruction (**E**) and three-dimensional CT scan (**F**).

**Figure 2:**
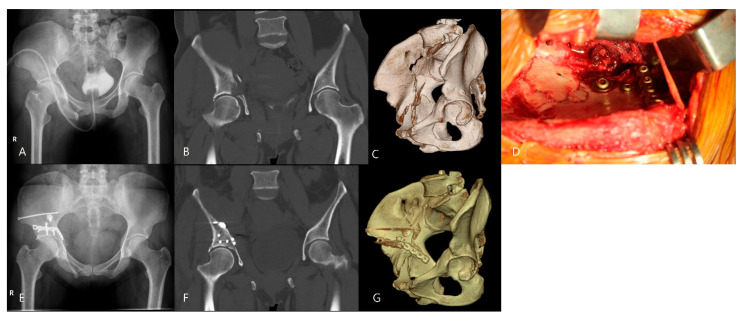
Standard anteroposterior radiograph (**A**) of a fracture of the right acetabulum. Coronal reconstruction CT scan (**B**) and three-dimensional CT scan (**C**) showing an incomplete anterior column fracture with a displaced quadrilateral plate fracture. Intraoperative photograph (**D**) showing anatomical reduction in the fracture and fixation with a lag screw and a spring plate. Postoperative anteroposterior radiograph (**E**) of the anterior column fracture stabilized using a supraacetabular lag screw and a spring plate. Postoperative sagittal reconstruction (**F**) and three-dimensional CT scan (**G**) showing anatomical reduction at the weight-bearing surface.

This retrospective case series was reviewed and approved by the Institutional Review Board of our institution (IRB no. DFH2004-01-001). Between 2006 and 2020, patients with an isolated incomplete high anterior column fracture of the acetabulum were included in this study. Since the introduction of our surgical technique, all consecutive patients with incomplete high anterior column fractures were consistently treated using this method; therefore, these patients were identified and analyzed through a retrospective chart review. A total of 28 patients were identified during the study period; among them, three patients were excluded due to loss to follow-up before 12 months, leaving 25 patients for final analysis. The diagnosis of incomplete high anterior column fractures and associated quadrilateral plate fractures was based on preoperative radiographic evaluation, including three standard radiographs (anteroposterior, iliac oblique, and obturator oblique views) and computed tomography (CT) scans, as well as intraoperative findings. On plain radiographs, disruption of the iliopectineal line with a fracture line extending superiorly toward the iliac wing was identified, while the posterior column remained intact. An incomplete high anterior column fracture was defined as a fracture in which the fracture line, when extended, would pass proximally to the anterior superior iliac spine, without complete disruption of the iliac crest on CT imaging (Figure 1 and Figure 2).

A quadrilateral plate fracture was suspected on plain radiographs when medialization of the femoral head was observed and was confirmed on CT imaging as a disruption of the medial wall of the acetabulum. All diagnoses were confirmed intraoperatively (Figure 2).

Displacement of the fracture >5 mm on a computed tomography (CT) scan was considered an indication for surgical treatment. Exclusion criteria were patients <16 years of age, follow-up period <12 months, open fractures, and pathological fractures.

Patient demographics, the mechanism of injury, associated injuries, time to surgical reconstruction, operation time, and postoperative complications were collected from the medical records. The clinical and radiographic results, according to Matta’s criteria, were evaluated at the final follow-up.

The quality of fracture reduction was assessed on a CT scan performed postoperatively. Further, it was graded as anatomical (displacement: 0–1 mm), imperfect (displacement: 2–3 mm), and poor (displacement: >3 mm) according to Matta’s criteria [8].

Radiographic outcomes at final follow-up were evaluated based on Matta’s radiological grading system: “excellent” indicated a normal appearing hip joint; “good” indicated mild changes with minimal sclerosis and joint narrowing (<1 mm); “fair” indicated moderate changes with joint narrowing (<50%); and “poor” indicated advanced degenerative changes [8]. Fracture healing was defined radiographically as the disappearance of the fracture line on anteroposterior and Judet views, and clinically as the ability to perform weight-bearing without pain.

Clinical outcomes were assessed using the modified Matta score, which consists of pain, walking ability, and range of motion (each scored from 1 to 6; total score range, 3–18). Scores were categorized as excellent (18), good (15–17), fair (13–14), and poor (<13) [8].

Postoperative complications such as infection, neurovascular injury, heterotopic ossification, and the development of osteoarthritis were also assessed.

Continuous variables were expressed as mean ± standard deviation and compared using the Mann–Whitney U test. Categorical variables were analyzed using Fisher’s exact test. A *p*-value < 0.05 was considered statistically significant. Statistical analyses were performed using SPSS software (version 24; IBM Corp., Armonk, NY, USA).

### 1.2. Operative Technique

The patient is placed in a supine position on a radiolucent operating table. The fracture is exposed through the anterior iliac approach adapted from the lateral window of the classic ilioinguinal approach [9]. The incision starts at the junction of the posterior and middle third of the iliac crest, curves toward the anterior superior iliac spine and ends 1–2 inches medial to the anterior superior iliac spine. The lateral femoral cutaneous nerve is exposed and protected. The iliopectineal fascia is detached from the iliopectineal eminence in some cases to place a longer plate and to insert an infra-acetabular screw. There are two types of incomplete high anterior column fractures: those with or without a displaced quadrilateral plate fracture. Both types are treated using a less invasive surgical technique of compression osteosynthesis through the anterior iliac approach, utilizing only the minimally modified lateral window of the ilioinguinal approach that was previously reported [9].

Incomplete anterior column fractures without quadrilateral plate involvement are reduced without osteotomizing the iliac crest using the following steps (Figure 1): First, the anterior column fracture is reduced by compressing the displaced anterior column using colinear clamps (Synthes, Zuchwil, Switzerland), which are applied from the lesser sciatic notch to the pelvic brim. Second, one or two lag screws (4.5 mm cortical screw) with a washer are inserted in the supraacetabular area to achieve interfragmentary compression. Third, a 4.5 mm under-contoured narrow limited contact dynamic compression plate (Synthes, Zuchwil, Switzerland) or a 4.5 mm reconstruction plate (Synthes, Zuchwil, Switzerland) is placed on the pelvic brim and fixed with two 4.5 mm cortical screws via the proximal holes of the plate placed on the intact ilium just lateral to the sacroiliac joint for additional compression and buttress of the anterior column fracture. The lag screws and the screws in the plate holes are tightened alternatively to achieve maximum interfragmentary compression without breaking the cortical bone. The plate is fixed additionally with one or two 4.5 mm cortical screws via the plate hole in the supraacetabular area, as in the lag screw technique. Fourth, an infra-acetabular screw is inserted to increase the fixation strength if possible.

In incomplete anterior column fractures with a displaced quadrilateral plate fracture, fracture reduction and osteosynthesis are conducted as follows (Figure 2): First, the anterior column fracture is reduced by compressing the displaced anterior column with a colinear reduction forceps. One or two lag screws (a 3.5- or 4.5 mm cortical screw or a 6.0 mm cancellous screw) are inserted in the supraacetabular area to achieve interfragmentary compression of the anterior column fracture. Second, the quadrilateral plate fracture is reduced with colinear reduction forceps. Third, an over-contoured spring plate made preoperatively with a 3.5 mm 7- or 8-hole reconstruction plate is placed on the quadrilateral plate and the supraacetabular area of the anterior column. The plate is pulled laterally with pointed reduction forceps to compress the quadrilateral plate. Three or four 3.5 mm cortical screws are inserted eccentrically via the plate holes, as in the lag screw technique, for additional compression and buttress of the anterior column and the quadrilateral plate.

### 1.3. Postoperative Rehabilitation

Continuous passive motion or active motion of the hip joint is started after acute pain is sufficiently reduced with analgesics, usually on postoperative day 3. Partial weight-bearing of a maximum of 20 kg is started at 3 weeks after surgery. Full weight-bearing ambulation is allowed at 6–8 weeks, depending on fracture healing and muscle strength.

## 2. Results

Twenty-five patients with isolated displaced incomplete high anterior column fractures of the acetabulum treated by compression osteosynthesis through the anterior iliac approach met the inclusion criteria (Table 1).

The mean age of the patients was 54.4 years (range, 20–82 years), and 21 patients were male. The mean time from injury to surgery was 5.2 days (range, 2–10 days). The mean surgical time (skin-to-skin) was 110 min (range, 75–160 min). The mean time to bone union was 10.2 ± 1.4 weeks (range, 8–14 weeks). The mean follow-up duration was 44.9 months (range, 12–142 months).

The causes of injury were falls from height (*n* = 14), motor vehicle accidents (*n* = 9), and ground-level falls (*n* = 2). Of the 25 incomplete high anterior column fractures, 13 had a displaced quadrilateral plate fracture, while the remaining 12 did not. Thirteen patients had associated injuries (Table 2).

The quality of fracture reduction was assessed as anatomical reduction in 22 patients, imperfect reduction in one patient because of incomplete reduction in the anteriorly displaced fracture fragment, and poor reduction in two patients because of failed reduction in the impacted articular fragment (Table 1). Clinical results were excellent in 19 patients and good in six patients; radiographic results were excellent in 22 patients and good in three patients (Table 1). All patients who achieved anatomical reduction showed excellent radiographic results. However, three of them showed good clinical results due to mild pain and reduced range of motion of the affected hip at the final follow-up. Among the three patients with non-anatomic reduction, two had small osteophytes of the femoral head, and one revealed mild narrowing of the joint space of the affected hip at the final radiographs.

Regarding the comparison between patients with and without a quadrilateral plate fracture, there were no significant differences between the two groups in terms of age, operation time, bone union time, transfusion rate, quality of reduction, or clinical and radiographic outcomes. Although no statistically significant differences were observed, all patients who did not achieve anatomical reduction or an excellent radiographic outcome had an associated quadrilateral plate fracture (Table 3).

The most common complication related to the surgical approach was an injury to the lateral femoral cutaneous nerve, which occurred in 13 patients (52%). Five of them were resolved entirely without treatment. The remainder partially improved but did not result in significant discomfort at the final follow-up. One patient had an injury to a small branch of the internal iliac artery during surgical procedures, which required pelvic packing and embolization of the artery. There were no patients with an injury to the femoral vessel or nerve. There were no cases of loss of reduction, postoperative infection, or heterotopic ossification.

## 3. Discussion

The current study demonstrated favorable clinical and radiographic outcomes using compression osteosynthesis without iliac crest osteotomy for displaced incomplete high anterior column fractures of the acetabulum. Moreover, this technique can be performed through a limited anterior iliac approach, potentially reducing surgical morbidity.

The clinical result of displaced acetabular fractures is strongly correlated with the accuracy of fracture reduction [1,2,3,10,11,12,13]. Therefore, open reduction and internal fixation to achieve anatomic reduction is a surgical strategy for displaced anterior column fractures. In line with this strategy, the necessity of osteotomizing the iliac crest to convert an incomplete fracture into a complete fracture in order to achieve anatomical reduction in displaced incomplete high anterior column fractures has been discussed [6,7].

However, in our experience, fracture reduction sometimes became more challenging after iliac crest osteotomy due to plastic deformation of the iliac wing. Once the incomplete fracture is converted into a complete fracture, restoration of the original anatomy may be difficult, particularly when the fragment has undergone plastic deformation. To overcome this issue, we began to reduce and stabilize these fractures without completing the incomplete iliac crest fracture. In addition, since the distal portion of the superior pubic ramus in high anterior column fractures does not involve the weight-bearing dome and therefore does not require anatomical reduction, these fractures can be adequately managed through a less invasive approach utilizing only the lateral window of the ilioinguinal approach.

In the present series, anatomical reduction was achieved in 22 of 25 patients. At the final follow-up, radiographic outcomes were excellent in 22 patients and good in three. While this study was not designed as a comparative study, these results are comparable to previously reported outcomes using the ilioinguinal, modified Stoppa, or pararectus approaches [14,15,16,17,18]. No statistically significant differences were detected between patients with and without associated quadrilateral plate fractures; however, this analysis was exploratory and likely underpowered due to the small sample size. Notably, all cases without anatomical reduction or excellent radiographic outcomes occurred in the quadrilateral plate fracture group, suggesting a potential trend. Among these, two cases showed poor reduction due to failure to reduce impacted dome fragments. Freude et al. emphasized that dome impaction should be carefully evaluated and addressed in the management of quadrilateral plate fractures [19]. Because the anterior iliac approach provides an extra-articular and relatively limited exposure, reduction in impacted articular fragments through the fracture line is technically challenging. Therefore, impacted dome fragments should be carefully identified preoperatively on CT scans and, if present, addressed through an additional iliac cortical window during surgery.

Regarding fixation, preservation of the incomplete iliac crest fracture simplifies the procedure and may shorten operative time, as no additional osteotomy or subsequent restabilization is required. The mean operative time of 110 min in this study appears to be shorter than that reported in previous studies using other anterior approaches, although direct comparison is limited by differences in patient populations and fracture characteristics [14,15,20,21,22]. Furthermore, the intact iliac crest provides inherent structural stability during reduction and fixation. However, because plastic deformation of the iliac wing may still be present, gradual and controlled compression should be applied while carefully monitoring the fracture gap to achieve sufficient interfragmentary compression and anatomical realignment.

The anterior iliac approach used in this study, which represents a minimal modification of the lateral window of the ilioinguinal approach, provides sufficient exposure for compression osteosynthesis in high anterior column fractures [9]. As this approach does not require surgical dissection of the middle and medial windows of the ilioinguinal approach, some complications related to more extensive anterior approaches may be reduced. However, complications were not negligible in this series. In particular, tension applied to the lateral femoral cutaneous nerve during this approach may still result in neuropraxia or sensory disturbance. This complication is consistent with previous reports of lateral femoral cutaneous nerve injury occurring in 12–57% of patients undergoing the ilioinguinal approach [2,3,17,23]. In our series, injury to the lateral femoral cutaneous nerve occurred in 13 patients (52%), and one case of vascular injury was observed. Care must be taken to prevent damage to the nerve by avoiding excessive stretching during surgical procedures.

All outcomes in this study were assessed at the final follow-up, and favorable clinical and radiographic results were consistently observed. Notably, among the eight patients with a follow-up of approximately 5 years or longer (≥59 months), seven achieved anatomical reduction, suggesting that the favorable outcomes were maintained over time.

This study has several limitations. First, it is a retrospective case series with a relatively small sample size. Second, we did not perform a direct comparison with other surgical techniques involving iliac crest osteotomy, and therefore, caution is required when interpreting comparisons with previously published studies. Third, the follow-up duration was heterogeneous among patients, which may have influenced the assessment of long-term outcomes, although favorable results were also observed in patients with longer follow-up periods. Given the low incidence of incomplete high anterior column fractures, a multicenter study with a larger patient population would be required to enable comparative analysis and to strengthen external validity.

A major strength of this study is that all procedures were performed by the senior author, who developed the technique, or by a surgeon trained and directly supervised by the senior author. This minimized inter-operator variability and ensured procedural consistency, allowing a more accurate assessment of the technical validity of the procedure itself.

## 4. Conclusions

Incomplete high anterior column fractures can be reduced and stabilized using compression osteosynthesis through the anterior iliac approach without completing the incomplete fracture by osteotomizing the iliac crest. In this case series, this technique demonstrated favorable radiographic and clinical outcomes. The use of a limited anterior approach may be associated with reduced surgical morbidity. Although a direct comparison was not performed, the outcomes observed in this study appear comparable to those reported in previous studies. Therefore, our surgical technique is a valuable addition to the existing techniques in treating high anterior column fractures of the acetabulum.

## Figures and Tables

**Table 1:** Demographic and clinical characteristics of the included patients.

Case Number	Age	Sex	Injury Side	Injury Mechanism	Quadrilate-ral Plate Fracture	Operation Time (min)	Trans-fusion	Bone Union (Weeks)	Follow-Up (Months)	Quality of Reduction	Final Results	
											Radiographic	Clinical
1	20	F	Right	fall from height (5th floor)	No	80	Yes	10	12	Anatomic	Excellent	Excellent
2	49	M	Right	motor vehicle accident	Yes	160	Yes	9	115	Anatomic	Excellent	Excellent
3	34	M	Right	fall from height (3 m)	Yes	95	No	10	128	Anatomic	Excellent	Excellent
4	31	F	Right	motor vehicle accident	Yes	110	Yes	12	65	Anatomic	Excellent	Excellent
5	31	M	Right	fall from height (2.5 m)	No	125	No	9	80	Anatomic	Excellent	Excellent
6	68	M	Right	motor vehicle accident	Yes	95	Yes	12	27	Anatomic	Excellent	Good
7	54	M	Left	fall from height (2 m)	Yes	145	Yes	8	36	Anatomic	Excellent	Excellent
8	67	M	Right	fall from height (1.5 m)	Yes	140	Yes	14	123	Anatomic	Excellent	Excellent
9	52	M	Right	fall from height (3 m)	No	140	Yes	10	16	Anatomic	Excellent	Excellent
10	53	M	Left	fall from height (3 m)	No	130	Yes	10	15	Anatomic	Excellent	Excellent
11	52	M	Right	fall from height (3 m)	No	85	No	10	13	Anatomic	Excellent	Excellent
12	69	M	Right	motor vehicle accident	No	75	Yes	10	12	Anatomic	Excellent	Excellent
13	67	M	Right	fall from height (1.5 m)	Yes	100	Yes	10	59	Anatomic	Excellent	Excellent
14	42	F	Right	motor vehicle accident	Yes	90	Yes	10	142	Anatomic	Excellent	Excellent
15	58	M	Right	motor vehicle accident	Yes	100	Yes	12	25	Anatomic	Excellent	Excellent
16	47	M	Right	fall from height (3 m)	Yes	115	Yes	12	17	Imperfect	Good	Good
17	68	M	Right	fall from height (ladder)	No	125	Yes	10	12	Anatomic	Excellent	Excellent
18	58	M	Right	fall from height (1.5 m)	Yes	130	Yes	10	64	Poor	Good	Good
19	82	M	Left	motor vehicle accident	No	125	Yes	8	31	Anatomic	Excellent	Excellent
20	52	M	Left	motor vehicle accident	No	100	No	10	16	Anatomic	Excellent	Excellent
21	37	M	Left	fall from height (4 m)	No	120	Yes	10	33	Anatomic	Excellent	Good
22	58	M	Left	motor vehicle accident	No	90	Yes	10	22	Anatomic	Excellent	Excellent
23	79	F	Left	ground-level fall	No	90	Yes	10	22	Anatomic	Excellent	Good
24	63	M	Right	fall from height (2 m)	Yes	105	Yes	8	12	Anatomic	Excellent	Excellent
25	71	M	Right	ground-level fall	Yes	80	Yes	10	27	Poor	Good	Good

**Table 2:** Associated injuries (including overlapped injury).

Associated Injuries	No. of Patients
None	12
Chest injury	3
Abdominal visceral injury	2
Brain injury	1
Spine injury	1
Extremity fracture	8

**Table 3:** Comparison between patients with and without quadrilateral plate fracture.

	Quadrilateral Plate Fracture (+) (*n* = 13)	Quadrilateral Plate Fracture (−) (*n* = 12)	*p*-Value
Age	54.5 ± 13.6	54.4 ± 16.8	0.985
Operation time (minutes)	112.7 ± 26.2	107.1 ± 23.4	0.554
Bone union (weeks)	10.5 ± 1.6	9.8 ± 1.1	0.150
Transfusion, *n* (%)	12 (92.3%)	9 (75.0%)	0.322
Anatomic reduction, *n* (%)	10 (76.9%)	12 (100%)	0.220
Excellent radiographic outcome, *n* (%)	10 (76.9%)	12 (100%)	0.220
Excellent clinical outcome, *n* (%)	9 (69.2%)	10 (83.3%)	0.650

Values are presented as mean ± standard deviation or number (%). Continuous variables were compared using the Mann–Whitney U test, and categorical variables were analyzed using Fisher’s exact test. A *p*-value < 0.05 was considered statistically significant.

## Data Availability

The data supporting this study are not publicly available due to patient privacy and ethical restrictions.
